# Mood Effects After Deep Brain Stimulation for Parkinson's Disease: An Update

**DOI:** 10.3389/fneur.2019.00617

**Published:** 2019-06-14

**Authors:** Ettore A. Accolla, Claudio Pollo

**Affiliations:** ^1^Neurology Unit, Department of Medicine, HFR – Hôpital Cantonal Fribourg and Fribourg University, Fribourg, Switzerland; ^2^Department of Neurosurgery, Inselspital, University Hospital Bern, University of Bern, Bern, Switzerland

**Keywords:** depression, hypomania, DBS, STN, GPi, basal ganglia

## Abstract

Depression in Parkinson's Disease (PD) is a prevalent and invalidating symptom. Deep brain stimulation (DBS) allows for an improvement of PD motor features, but its effects on mood are difficult to predict. Here, we review the evidence regarding mood effects after DBS of either subthalamic nucleus (STN) or globus pallidus pars interna (GPi). Different influences of multiple factors contribute to impact the neuropsychiatric outcome after surgery. Psychosocial presurgical situation, postsurgical coping mechanisms, dopaminergic treatment modifications, and direct effects of the stimulation of either target are all playing a distinct role on the psychological well-being of patients undergoing DBS. No clear advantage of either target (STN vs. GPi) has been consistently found, both being effective and with a favorable profile on depression symptoms. However, specific patients' characteristics or anatomical considerations can guide the neurosurgeon in the target choice. Further research together with technological advances are expected to confine the stimulation area within dysfunctional circuits causing motor symptoms of PD.

## Depression and Parkinson's Disease (PD)

Depression in PD is a common finding, and has an important impact on patient's quality of life (QoL) (1). Published prevalence estimates vary depending on the population studied, but significant depressive symptoms occur in around 35% of patients (2). The impact of depression on patients' well-being can hardly be overestimated. It appears to be more distressing for patients and their families than motor symptoms (3). Treating depression have beneficial effects also on motor performance (4) and conversely depressive symptoms are among the stronger predictors of initiation of dopaminergic therapy (5).

While depression is among the most common non-motor symptoms of PD, its pathophysiology is incompletely understood. As in any chronic illness, a reactive depression is observed after the diagnosis is communicated to the patient, related to fears about PD and actual or perceived disability. However, mood symptoms tend to occur 4–6 years before the motor symptoms, and most often predate the established diagnosis, indicating that neurobiological factors are more determinant than psychosocial ones (6). According to the Braak hypothesis (7), alfa-synuclein deposition and corresponding neurodegeneration in PD begins in non-dopaminergic brainstem structures, including locus coeruleus and raphe nucleus. A decrease of endogenous production of serotonin have been documented both *post-mortem* (8) and *in-vivo* (9), supporting the notion of an organic origin to depression in PD. The involvement of other neurotransmitters, including noradrenaline and dopamine, is testified by the positive effects on mood of dopamine agonists and stimulants such as methylphenidate (10, 11). Further, multiple neuroimaging studies describe anatomical and functional differences among PD patients with depression and without depression: the most consistent evidence shows reduced activity and possibly gray matter density in the frontal lobe and other limbic cortical and subcortical structures (12).

## Mood Impact of DBS

The surgery for DBS most often follows a long path of physical deterioration as a consequence of advancing PD. From the patient's perspective, the decision to accept such an invasive procedure is often a difficult one, carefully pondered, sometimes perceived as a last resort before losing an acceptable quality of life. It is rightfully considered a major event by patients. Even if they are duly and fully informed about the limitations of DBS, the procedure is often accompanied by exaggerated expectations, that match the (overestimated) perceived surgical risk. As a consequence, patients might fail to cope with the numerous hurdles they may encounter after the surgery. One of these hurdles is represented by the important modifications in familial and social relationships, that occur as a result of the overall improvement of the motor symptoms. Family members and caregivers might interpret the striking reduction of tremor and dyskinesias as the relieving end of their relative's need for care, after they underwent the “miraculous” surgery. On the other side, the patient could suddenly loose his “disease label” that had become part of his own identity, and informed most of his social relationships. Hindrances from non-motor symptoms are often not adequately recognized and understood by caregivers. Couples can be particularly put at strain: after years of daily routine revolving around the medication timetable, the anticipation of motor fluctuations and dyskinesias, major changes can intervene, and modify intensely bonding dynamics, centered on the disease (13–15).

Accounting for these social and psychological factors is of the highest importance during the pre-operative evaluation for DBS, and must be included in the multidisciplinary team discussions.

In general, the DBS procedure has a positive effect on mood aggregate scales, independently from the chosen target (16, 17); this was confirmed by a recent meta-analysis, which found a small and moderate reduction in depression symptoms after STN-DBS or GPi-DBS, respectively (18). Interestingly, there is an important heterogeneity in the observed pre vs. post-surgical modifications in depression scores, which seems to be modulated by two related factors: disease duration and UPDRS score. Perhaps not surprisingly, the greater benefit on depression scores was observed in those patients who had endured PD for more years, and suffered from a greater functional impairment. It must be underscored however, that depression severity scores do not necessarily correlate with these two factors. A more plausible interpretation stemming from this meta-analysis is that when the disease is advanced the dramatic improvement of motor symptoms and levodopa complications after DBS has a notable effect on depression symptoms, independently from the starting depression severity.

The effects of DBS on brain neurochemistry (including dopaminergic and non-dopaminergic systems) is an interesting topic of investigation, still insufficiently explored: rodent experiments suggest that DBS might in part act by modulating 5HT and NA release. An influence of DBS on limbic circuits mediated by indirect neurotransmitter release cannot be excluded (19, 20).

### STN-DBS Effects on Mood

STN is the most frequently used target for DBS. Its positive effects are broad and include tremor, rigidity, bradykinesia reduction, but also gait and balance improvement, when levodopa sensitive. Even if the effects on mood have been quite extensively studied, the literature reports conflicting results, probably due to differences in cohorts or assessment methods. Early reports found a relatively high risk of suicide among patients treated by DBS (21–24), mostly (although not only) in patients with a previous depression history. The absolute numbers of post-operative suicides qualify it as a rare event, and as such the cohorts are underpowered to conclude for an actual increase of suicides or suicidal ideation among STN-DBS patients. Moreover, multiple and diverse factors can play a role in the observed apparent increase of the suicide rate. Among others, a selection bias could exist, so that patients least accepting their disease are more willing to undergo DBS. This population would be more prone to disappointment when DBS fails to meet their expectations. Expectations should not be unrealistic, and the role of the caring team is to assure that patients fully understand the risks and the likely benefits from DBS, particularly underscoring that DBS will not stop the neurodegenerative disease to progress.

In face of these early findings, the formal assessment of depressive symptoms showed positive effects of STN-DBS (25–28). The outcome on mood symptoms is highly variable and likely depends on multiple factors, that underscore how patient's selection is crucial for a positive outcome (29).

#### Dopamine-Withdrawal Syndrome

STN-DBS allows for an important reduction of dopaminergic medication, if not for a complete withdrawal. Neuronal degeneration in PD involves also mesolimbic dopaminergic cells in the ventral tegmental area, which project to ventral striatum and orbito-frontal regions that are involved in non-motor functions, which are central in the brain circuitry of motivation and reward. It is widely believed that a complete interruption of dopaminergic medication, even if allowed from a motor point of view, can be responsible for the progressive appearance of a postoperative apathy and anhedonia, and eventually to the appearance or worsening of depressive symptoms (25, 30). This crucial aspect must be considered during the early and delayed postoperative phase, as apathy and its consequences can appear slowly and progressively. Their correct assessment and management can prevent depressive states and ultimately improve the overall outcome of STN-DBS (30, 31).

The direct induction of neuropsychiatric symptoms by STN-DBS is well documented, particularly for the induction of hypomanic or manic states, which can be reversed by modifying the stimulation parameters. These effects are thought to arise following the stimulation of sub-areas of the STN involved in limbic circuits, as first suggested by Krack et al. after observing a stimulation-induced “mirthful laughter” reaction (32). Multiple lines of evidence document the existence of a functional specialization within the STN, and basal ganglia structures in general (33–37).

Cortico-subcortico-cortical loops seem to maintain a certain degree of functional segregation within each node, by which sub-areas within each structure can be identified as preferentially connected to cortical and subcortical regions processing similar information. Within this general principle of organization, the STN seem to play a pivotal role, and host the integration of different functional circuits (34). The posterior-dorsal STN is the target of DBS for PD, as it is thought to be a node of the motor circuit (with an overall antikinetic function). More anteriorly and ventrally however, the STN has a stronger connectivity with cortical areas involved in cognitive and emotional (“limbic”) functions (38): this is considered as being the anatomical substrate underlying undesired neuropsychiatric effects of DBS. The induction of hypomanic states has been reported most consistently following ventral STN stimulation: patients suffer from an excited hyperactivity, reduce their sleep time, engage in reward-seeking behaviors including unnecessary buying, with heavy consequences on familial or working relationships. The fact that these behavioral disturbances can be promptly reversed by modifying stimulation parameters, supports the notion of a direct role of the stimulation of non-motor STN subareas. The most effective management of hypomanic manifestations imply shifting the stimulation to more dorsal contacts, bringing indirect evidence on the ventral location of the “limbic” STN.

## GPI vs. STN-DBS Mood Effects

The globus pallidus internus (GPi) has been used as a stimulation target for Parkinson's Disease since the early days of DBS, due to its recognized benefits on motor symptoms (39, 40). However, STN has been consistently considered the preferred target, based on evidence from early trials not specifically designed (41) or weighted (42) for a comparison among the two stimulation targets. Eventually, most centers have been proposing almost exclusively STN as a DBS target for advanced PD, resulting in an accumulation of knowledge on the benefits and side effects of STN stimulation, leaving GPi-DBS comparatively less investigated. As an end result, less evidence is available about the psychiatric effects of GPi-DBS in PD. Some early assumptions—while possibly correct—have skewed patient selection, further propagating the publication bias. Most recently, at least three randomized clinical trials comparing GPi to STN have been conducted, specifically addressing the differential therapeutic profiles of the two stimulation targets (16, 43, 44). The trials are substantially concordant in describing a similar benefit on motor symptoms from DBS of either stimulation target. When analyzing motor sub scores however, some slight differences emerge—mostly with no statistical significance—and might be considered in the pre-implantatory phase. Among the cardinal PD motor features, rigidity, and bradykinesia could possibly respond better to STN than GPi stimulation; levodopa responsive gait and balance issues appeared to improve better with GPi stimulation in one of the trials, which also showed a trend toward more pronounced and sustained dyskinesias reduction after GPi than STN stimulation (45). All trials show a greater medication reduction after STN than GPi-DBS, which is sustained at 3 years follow-up (46).

Regarding neuropsychiatric effects, GPi-DBS seems to have a lesser impact than STN-DBS (45). This notion however is based on a limited number of well-conducted trials, and some confounding factors have to be taken in consideration. For instance, Anderson et al report early post-operatory anxiety as being more frequent after STN-DBS (47), a group in which medication reduction was more abrupt and important. A dopaminergic-withdrawal syndrome (30) could therefore be held responsible, and could have been prevented by slowly tapering down the medication (48). The COMPARE trial (16) assessed mood disorders through the Visual Analog Mood Score (VAMS), and did not find differences among STN and GPi in the compound score. However, the item “angry” was higher for the STN-operated patients. This was observed as well by Weintraub (49), who describe that “GPi patients were happier, less angry or bitter and less tired compared with STN patients.” In the same publication, authors could not confirm a higher risk of suicide among DBS patients, either implanted in the STN or GPi.

## Management of Depression in the Preoperative and Postoperative Phases

The existence of depressive symptoms is not *per se* a contraindication to DBS surgery. However, ongoing severe depression, psychotic symptoms, and suicidal ideation should be considered absolute contraindications as they might worsen and increase suicidal risk, particularly in the first year after surgery (50–52). Less evidence is available regarding severe depressive patients who were eventually stabilized by psychotherapy and medication, months or years prior to undergoing DBS: a trend toward a slightly worse motor and mood outcome has been described, but this certainly does not constitute an absolute contraindication to surgery (53). In any case, most groups and guidelines support the recommendation of a thorough psychiatric assessment before DBS surgery, and of a careful post-operative follow-up. Of note, the post-operative psychiatric assessment should not be limited to the immediate post-operative period, as the occurrence of apathy, for instance, peaks at around 4 months after surgery, often accompanied by depressive symptoms (30). Particularly after STN-DBS, which allows for a steeper reduction of dopaminergic medication, dopamine withdrawal symptoms should be prevented, when possible favoring the continued treatment with dopamine agonists (31).

## Neuroanatomical and Neurosurgical Considerations

The advent of DBS has offered an unprecedented opportunity for *in-vivo* investigation of the functional organization of basal ganglia. This knowledge has been growing together with clinical observations on patients undergoing DBS, and has driven further the development and the refining of this remarkable neuromodulation treatment. The occurrence of cognitive, behavioral and mood effects after DBS have been key in updating models of basal ganglia functioning, that help now predict the outcome of the procedure. From a neuroanatomic point of view, the large spectrum of effects observed after DBS confirmed some previous notions on the basal ganglia organization, but also exposed the limits of excessively schematizing models. The most influential model of basal ganglia organization (54, 55) proposed the existence of strictly segregated cortico-subcortico-cortical loops, each originating and terminating in distinct prefrontal cortical areas. Accordingly, circuits processing motor, cognitive, and emotional information would be kept segregated in basal ganglia nuclei, within subareas with a specific functional specialization. Accumulating evidence strongly suggests that there is substantial overlap of functional subareas, underpinning information exchange and integration (33, 34, 36, 38). This might partially explain the rich interaction among mood and motor symptoms (5) in PD, and most likely the occurrence of hypomanic manifestations after stimulation of dorso-lateral STN, which is considered as having a predominant motor function (25, 56). The distribution of cortico-STN projections in the primate (38) and human brain [as studied by DTI MRI-tractography (33, 34)] suggests that wrong positioning of the stimulating contact is not necessary to modulate non-motor parts of STN (57). Nevertheless, several studies were able to show that patients presenting postoperative hypomania were stimulated too ventrally, mostly within the anatomically defined limbic STN (56–58), and that shifting stimulation to more dorsal contacts was effective in reducing the neuropsychiatric symptoms.

The GPi is also involved in multiple non-motor functions, and probably has a topographical organization mirroring what observed in STN, with an antero-posterior and ventro-dorsal gradient of connectivity (limbic->associative->motor). This has been demonstrated thanks to tracing experiments in non-human primates (59), confirmed in DTI imaging studies (60). It must be underscored however that GPi does not receive direct cortical inputs; this parcellation is based mainly on the distribution of connections to striatum. Therefore, the exact topography of GPi sub territories is less clear than for the STN. Nevertheless, the relative volume of the motor GPi to be targeted by DBS, is definitely greater than the motor STN. Therefore, the probability of stimulating current spreading to non-motor territories is probably lower (16). In that respect, this might represent an advantage of the GPi over the STN when choosing the stimulating target based on the theoretical risk of non-motor undesired side effects.

The advent of segmented (directional) electrodes have shown to increase the therapeutic window for controlling motor symptoms (61, 62). This new technology provides a unique opportunity to investigate and refine anatomo-clinical correlations for non-motor symptoms as well, as these investigations can be performed not only in the vertical plane (Z axis), but also in the horizontal plane (X and Y axes). A better localization of non-motor areas in the STN should prevent the appearance of related symptoms. Recent advances in so-called “adaptive stimulation” are also promising in reducing undesired DBS side effects related to stimulation of non-motor STN (or GPi) areas. Based on recorded neurophysiological activity, the stimulation intervenes only when detecting neurophysiological activity linked with the motor akinesia, and delivers an intermittent stimulation tailored on patients' needs (63, 64).

## Concluding Remarks

Careful patient selection, an experienced neurosurgeon, and adequate peri-operative management of mood disturbances make DBS a safe treatment in terms of expected neuropsychiatric outcomes. A causative role for the stimulation in triggering or worsening psychiatric diseases appear unlikely, which is overall reassuring. Hence, there is insufficient evidence to conclude for an advantage of a stimulation target solely based on the expected effects on depressive symptoms. We do recommend however to carefully assess the neuropsychiatric profile of each patient and let it inform the decisional process when considering both STN and GPi as stimulation targets. Specific preoperative symptoms (apathy, bipolar traits, anxiety, dopamine dysregulation syndrome among others) could tilt the balance toward any of the two targets, possibly improving DBS outcome for a given patient. Further technological advances are expected to improve surgical targeting and optimize stimulation methods, allowing for a timely modulation restricted to the dysfunctional circuits causing PD symptoms.

## Author Contributions

EA made a review of the literature and wrote the manuscript. CP reviewed the manuscript.

### Conflict of Interest Statement

The authors declare that the research was conducted in the absence of any commercial or financial relationships that could be construed as a potential conflict of interest.
